# Temperature as a Key Modulator: Investigating Phosphorylation Patterns of p.Asn666 *PDGFRB* Variants and Their Role in Downstream Signaling

**DOI:** 10.1155/humu/6664372

**Published:** 2025-04-22

**Authors:** Titas Gladkauskas, Ileana Cristea, Roya Mehrasa, Jean-Baptiste Demoulin, Bjørn Tore Gjertsen, Ove Bruland, Eyvind Rødahl, Cecilie Bredrup

**Affiliations:** ^1^Department of Clinical Medicine, University of Bergen, Bergen, Norway; ^2^Bergen Center for Medical Stem Cell Research, University of Bergen, Bergen, Norway; ^3^Department of Ophthalmology, Haukeland University Hospital, Bergen, Norway; ^4^De Duve Institute, University of Louvain, Brussels, Belgium; ^5^Department of Internal Medicine, Haukeland University Hospital, Bergen, Norway; ^6^Department of Medical Genetics, Haukeland University Hospital, Bergen, Norway

**Keywords:** germline activating mutations, PDGFRB, receptor tyrosine kinase, temperature sensitivity, thermokinetics

## Abstract

Four different amino acid substitutions have been reported at the p.Asn666 position in platelet-derived growth factor receptor *β* (PDGFR*β*): p.Asn666Lys, p.Asn666Tyr, p.Asn666Ser, and p.Asn666His. All four substitutions result in strikingly different phenotypes, ranging from somatic infantile myofibromatosis in p.Asn666Lys and ocular pterygium–digital keloid dysplasia in p.Asn666Tyr to a severe form of Penttinen syndrome in p.Asn666Ser, while p.Asn666His is associated with a complex phenotype characterized by debilitating hand and foot contractures and facial coarseness. Here, we show that the p.Asn666Lys, p.Asn666Tyr, and p.Asn666His substitutions result in increased total PDGFR*β* phosphorylation at 32°C compared to 37°C. All four substitutions exhibit distinct activation patterns of specific PDGFR*β* tyrosine residues at both temperatures, indicating a unique activation of each variant. The temperature effect on downstream signaling is present across all substitutions, resulting in substitution-specific downstream signaling at both 37°C and 32°C. This complex interplay of downstream signaling proteins could be important for the clinical manifestations of p.Asn666 *PDGFRB* variants. Furthermore, variant-specific overactivation of tyrosine residues and downstream signaling at 32°C emphasize the importance of temperature as an environmental factor in the pathogenesis of this diverse group of disorders.

## 1. Introduction

The *PDGFRB* gene encodes the platelet-derived growth factor receptor beta (PDGFR*β*) protein, a transmembrane receptor tyrosine kinase (RTK) [1]. Upon platelet-derived growth factor (PDGF) binding, dimerization of the kinase occurs, followed by phosphorylation of the tyrosine residues located in the intracellular domain [2, 3]. The phosphorylated tyrosine residues facilitate the binding of downstream signaling molecules [4, 5]. The cascade of events initiated by phosphorylation of PDGFR*β* ultimately affects gene expression and modulates cell proliferation, differentiation, and motility [1, 4]. Dysregulation of the PDGFR*β*/PDGF signaling, usually through somatic mutations or fusion with other genes, has been associated with several human disorders including cancers [6–9]. In addition, several pathogenic *PDGFRB* germline variants have been reported [10].

Gain-of-function germline variants, reviewed by Guérit and colleagues [10], have been linked to several different clinical conditions including infantile myofibromatosis, Kosaki overgrowth syndrome (KOGS), premature aging syndrome, Penttinen type (Penttinen syndrome), and ocular pterygium–digital keloid dysplasia (OPDKD). The *PDGFRB* variants p.Trp566Arg and p.Pro584Arg located in the juxtamembrane region are linked to KOGS [11–16]. The variants associated with familial cases of infantile myofibromatosis include p.Pro560Leu and p.Arg561Cys [17–19] located in the juxtamembrane region. Variants p.Val6665Ala and p.Asn666Ser, located in the kinase domain, are associated with Penttinen syndrome [20, 21]; p.Asn666Tyr is associated with OPDKD [22]; and p.Asn666His is reported in a single individual, with clinical manifestations described below [23]. These are all constitutively active, ligand-independent variants. One ligand-dependent activating variant has been reported in the transmembrane domain, namely, p.Ser548Tyr, linked to corneal vascular overgrowth in otherwise healthy individuals [24]. In the extracellular domain, the p.Ser493Cys variant has been described in one individual with KOGS [16]. In contrast, loss-of-function variants in *PDGFRB* have been associated with primary familial brain calcifications. These variants have been observed across all regions of the receptor [25–28].

Intriguingly, the five different known variants of p.Asn666 are associated with strikingly different clinical conditions. A summary of clinical data is presented in Table S1. Briefly, Cheung and coworkers [19] described the p.Asn666Lys variant in a patient with somatic infantile myofibromatosis (the patient also had a germline p.Arg561Cys alteration). This variant was later confirmed in other sporadic infantile myofibromatosis cases, both in combination with the p.Arg561Cys variant and alone [29]. Similarly, a recently identified variant associated with somatic infantile myofibromatosis includes the p.Asn666Thr and p.Ile564_Val572del mutations in *PDGFRB* [30]. The pathogenicity of this variant remains unconfirmed and was not investigated in this study. The p.Asn666His variant was found in one patient with a complex congenital syndrome with cranial, hand, dental, and growth abnormalities [23]. Furthermore, a recurrent germline p.Asn666Ser variant has been linked to a severe form of Penttinen syndrome [21, 31], a highly debilitating condition characterized by lipodystrophy, acro-osteolysis, chronic ulcers in limbs, and corneal vascularization. More recently, a p.Asn666Tyr variant has been described, associated with OPDKD [22]. This condition manifests as corneal vascular overgrowth in early childhood later followed by keloid formation on digits. While this substitution in patient fibroblasts gives near normal PDGFR*β* activation at the core body temperature of 37°C, at the lower physiological temperature of the cornea (32°C), ligand-independent overactivation of PDGFR*β* and downstream signaling proteins were found [22]. This can explain why only lower temperature parts of the body (cornea and digits [32, 33]) are affected. Also, in p.Asn666Ser fibroblasts, at 32°C, upregulation of p-STAT1, a downstream signaling protein of PDGFR*β*, was observed, indicating partial temperature sensitivity also of this *PDGFRB* variant [22].

To understand how p.Asn666Lys, p.Asn666Ser, p.Asn666His, and p.Asn666Tyr variants can lead to so different clinical conditions, we have investigated the phosphorylation of specific tyrosine residues in PDGFR*β* and downstream signaling proteins with a particular focus on the effects of physiologically reduced temperature (32°C) and ligand stimulation. We show that all four p.Asn666 *PDGFRB* variants exhibit distinct phosphorylation patterns both at 32°C and when stimulated with PDGF. All four variants are temperature sensitive, with increased activation at 32°C. Each p.Asn666 *PDGFRB* variant thus displayed a unique pattern of activation.

## 2. Materials and Methods

### 2.1. Transduction of Cells

Immortalized fibroblasts (CRL-4001, ATCC, Manassas, VA) were grown in Dulbecco's modified eagle medium (DMEM) from Gibco (Thermo Fisher Scientific, Waltham, MA) containing 10% fetal calf serum (Biosera, Cholet, France), penicillin, streptomycin (Euroclone, Siziano, Italy), and glutamine (Sigma-Aldrich, Saint Louis, MO). The construction of transgenic cells was performed as previously described [21]. In brief, a murine retroviral vector containing the human *PDGFRB* open-reading frame (NM_002609.3) was obtained from VectorBuilder (Cyagen Biosciences, Santa Clara, CA). The *PDGFRB* c.wt, c.1996A>T p.(Asn666Tyr), c.1997A>G p.(Asn666Ser), c.1996A>C (p.Asn666His), and c.1998C>A (p.Asn666Lys) variants were introduced using the QuikChange Site-Directed Mutagenesis Kit (Catalog #200518, Agilent Technologies, Sydney, Australia). Virus production was achieved by transfecting Phoenix-AMPHO packaging cells (CRL-3213, ATCC). Two days after transfection, the medium was harvested, and immortalized fibroblasts were transduced. Two days postinfection, stably transduced cells were selected by adding 1 *μ*g/mL puromycin (InvivoGen, San Diego, CA).

### 2.2. Cell Culture, Lower Physiological Temperature of the Cornea (32°C), and PDGF Stimulation

Transduced immortalized fibroblasts were seeded in 6-well plates. For temperature sensitivity experiments, cells were grown until 80% confluent, serum-starved for 16 h, and then either kept at 37°C or incubated for indicated time points at the physiological corneal temperature of 32°C [32]. Cells were stimulated with PDGF from human platelets (P8147-5UG, Merck KGaA, Darmstadt, Germany) 10 ng/mL in 4 mM HCl containing 0.1% BSA, for 20 min or with 4 mM HCl containing 0.1% BSA alone as a control. According to the manufacturer, the PDGF isolated from the platelets consists of approximately 70% PDGF-AB, 20% PDGF-BB, and 10% PDGF-AA. Cells were harvested using lysis buffer consisting of 1% NP-40, 20 mM Tris (pH 8.0), 137 mM NaCl, 10% glycerol, 2 mM EDTA, 1 mM activated sodium orthovanadate, 10 *μ*g/mL aprotinin (#4139, Tocris, Abington, United Kingdom), and 10 *μ*g/mL leupeptin (leupeptin hemisulfate, #1167, Tocris) and stored at −80°C until immunoblot or ELISA analysis was performed.

### 2.3. ELISA and Immunoblot Analysis

ELISA and immunoblot analysis were performed as previously described [21]. In brief, ELISA analysis was conducted using a DuoSet IC PDGFR*β* kit (# DYC1767-2, R&D Systems, Minneapolis, MN) following the guidelines provided by the manufacturer. In this kit, the total phosphorylation level of PDGFR*β* is measured using an immobilized antibody binding PDGFR*β* followed by an HRP-conjugated antibody against phosphorylated tyrosine.

For immunoblot analysis, protein separation was carried out using a high-resolution gel system (4–12% NuPAGE Novex Bis-Tris Gel; Life, Carlsbad, CA) following the manufacturer's instructions and transferred to nitrocellulose membranes (Bio-Rad, Hercules, CA). Membranes were blocked with 5% nonfat dry milk (Bio-Rad), 1% glycine, and 1% BSA in PBS-T buffer (standard PBS with 0.05% Tween 20). They were incubated overnight at 4°C with primary antibodies from Cell Signaling Technology (Danvers, MA) used at recommended dilutions (catalog numbers in brackets): phospho-Tyr701-STAT1 (#7649), STAT1 (#9172), phospho-Tyr705-STAT3 (#9145), STAT3 (#9132), phospho-Tyr641-STAT6 (#56554), STAT6 (#5397), phospho-Ser473-AKT (#4060), phospho-Thr308-AKT (#4056), AKT (#4691), phospho-Tyr783-PLC*γ*1 (#2821), PLC*γ*1 (#5690), phospho-PDGF Receptor *β* (Tyr740) (#3168), phospho-PDGF Receptor *β* (Tyr751) (#4549), phospho-PDGF Receptor *β* (Tyr771) (#3173), phospho-PDGF Receptor *β* (Tyr1009) (#3124), phospho-PDGF Receptor *β* (Tyr1021) (#2227), PDGF Receptor *β* (#3169), antirabbit IgG, and HRP-linked antibody (#7074). To control for equal loading, an anti-GAPDH antibody (#G99545, Sigma-Aldrich) was used. Chemiluminescence was detected using the ChemiDoc Touch Imaging System (Bio-Rad). ImageJ software was used for the relative quantification of immunoblot band intensity.

### 2.4. Statistical Analysis and Reproducibility

One-way ANOVA, followed by Dunnett's multiple comparisons test, and two-way ANOVA, followed by Šídák's multiple comparisons test, were performed for statistical analysis to compare total PDGFR*β* phosphorylation levels measured with ELISA. All results were replicated in at least three independent experiments. Representative images are shown in the figures.

## 3. Results

### 3.1. The p.Asn666Tyr, p.Asn666Ser, p.Asn666His, and p.Asn666Lys PDGFR*β* Substitutions Cause Ligand-Independent PDGFR*β* Activation

**Figure 1:**
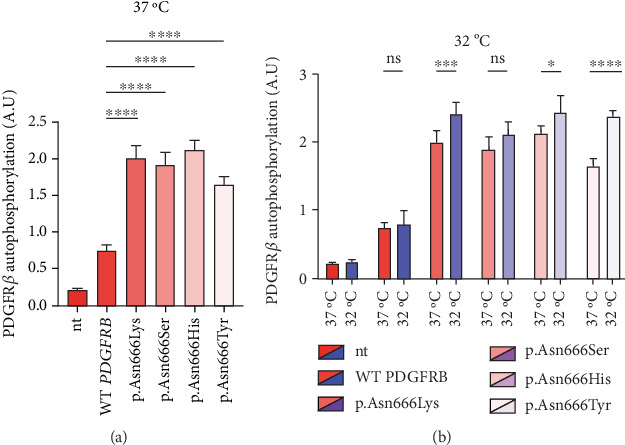
(a) All PDGFR*β* substitutions (p.Asn666Lys, p.Asn666Ser, p.Asn666His, and p.Asn666Tyr) increase basal phosphorylation at 37°C. (b) Exposure to 32°C for 6 h further increases phosphorylation in the p.Asn666Tyr, p.Asn666Lys, and p.Asn666His substitutions. Total phosphorylated PDGFR*β* levels were measured by ELISA. Nontransduced immortalized fibroblasts were referred to as “nt,” while fibroblasts transduced with *PDGFRB* wild-type vector were denoted as “WT *PDGFRB*.” (a) One-way ANOVA with Dunnett's test compared wild-type *PDGFRB* and variants at 37°C (⁣^∗∗∗∗^*p* < 0.0001). (b) Cells were incubated at 32°C or 37°C, and two-way ANOVA with Šídák's test compared phosphorylation between temperatures for each variant (⁣^∗^*p* < 0.05;^∗∗∗^*p* < 0.001;^∗∗∗∗^*p* < 0.0001).

**Figure 2:**
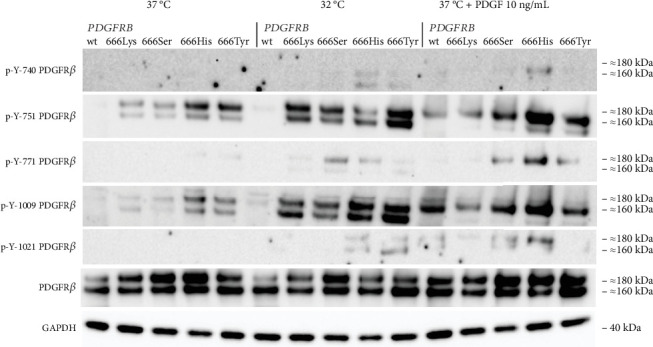
Effect of reduced temperature (32°C) and ligand stimulation on PDGFR*β*-specific tyrosine residue phosphorylation. Representative immunoblots showing phosphorylation of PDGFR*β* at p-Y-740, p-Y-751, p-Y-771, p-Y-1009, and p-Y-1021. At 37°C, p.Asn666Lys and p.Asn666Ser variants exhibited nearly identical signal intensity, whereas p.Asn666His and p.Asn666Tyr showed a distinct pattern. At 32°C, all variants exhibited a variant-specific increase in phosphorylation. PDGF stimulation (10 ng/mL) altered phosphorylation patterns, increasing the upper PDGFR*β* band, likely representing the fully mature, membrane-associated receptor. Full immunoblots, also overexposed, are shown in Figures S1 and S2.

In this experiment, immortalized fibroblasts were employed, which had been stably transduced with wild-type *PDGFRB* (wt), or with vectors expressing p.Asn666Lys, p.Asn666Ser, p.Asn666His, and p.Asn666Tyr substitutions. All four substitutions resulted in increased basal phosphorylation levels of PDGFR*β* compared to wild type at 37°C (2.66, 2.51, 2.89, and 2.2 folds, respectively) (Figure 1). After exposure to 32°C for 6 h, p.Asn666Tyr, p.Asn666Lys, and p.Asn666His showed increased PDGFR*β* phosphorylation. The p.Asn666Ser variant showed a trend to higher PDGFR*β* phosphorylation at 32°C; however, this was not statistically significant (Figure 1). In immortalized fibroblasts, similar levels of unphosphorylated PDGFR*β* were observed, indicating comparable levels of PDGFR*β* expression (Figure 2).

### 3.2. Phosphorylation of Specific PDGFR*β* Tyrosine Residues Upon Lower Physiological Temperature (32°C) and PDGF Stimulation

To further investigate the phosphorylation pattern of PDGFR*β*, we specifically examined the tyrosine residues p-Y-740, p-Y-751, p-Y-771, p-Y-1009, and p-Y-1021 (Figure 2). The four *PDGFRB* p.Asn666 variants exhibited increased phosphorylation of p-Y-751 and p-Y-1009 compared to the wild type. The p.Asn666His and p.Asn666Tyr variants showed the highest levels of phosphorylation of these tyrosine residues. A small amount of phosphorylated Y-771 was seen in p.Asn666His and p.Asn666Tyr. We did not detect any phosphorylation of Y-740 and Y-1021. Upon exposure to 32°C, we observed increased phosphorylation of all five PDGFR*β* tyrosine residues. Notably, at 32°C, the strongest signal remained for the Y-751 and Y-1009 residues in all four variants, while cells with p.Asn666Ser and p.Asn666His had the strongest phosphorylation of the Y-771 residue. The strongest phosphorylation of the Y-740 residue at 32°C was observed in the p.Asn666His variant. The most prominent phosphorylation of the Y-1021 residue was observed for the p.Asn666His and p.Asn666Tyr variants. At 32°C, a trend for increased signal intensity was observed in both the lower (160 kDa) and the upper (180–190 kDa) bands (Figure 2) (Figure S2).

We also examined the effect of ligand stimulation. After 20 min of incubation with 10 ng/mL of PDGF, a different pattern was seen. The most noticeable was the upregulation of the upper band (180–190 kDa) of PDGFR*β*. This was observed for all the p.Asn666 substitutions, as well as for the wild-type PDGFR*β* (Figure 2). As in the temperature experiments, the Y-751 and Y-1009 residues exhibited the highest phosphorylation levels, including the wild type. Among all p.Asn666 substitutions, p.Asn666His consistently displayed the strongest phosphorylation levels across all five phosphorylation sites, while p.Asn666Lys showed the weakest response to PDGF stimulation (Figure 2).

### 3.3. Specific Downstream Signaling Protein Changes at 37°C and 32°C for the p.Asn666Tyr, p.Asn666Ser, p.Asn666His, and p.Asn666Lys Amino Acid Substitutions

**Figure 3:**
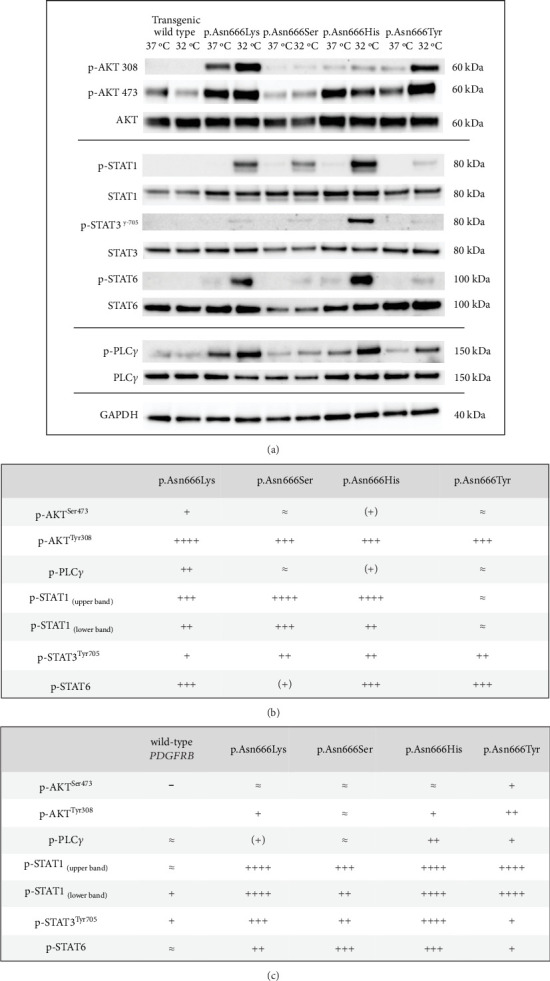
Effects of lower physiological temperature (32°C) on PDGFR*β* downstream signaling. (a) Immunoblots showing temperature-dependent changes in p-AKT-Ser473, p-AKT-Thr308, p-STAT1, p-STAT3-Tyr705, p-STAT6, and p-PLC*γ*1, in immortalized fibroblasts expressing the four p.Asn666 variants. GAPDH served as a loading control. Full immunoblots are shown in Figure S3. (b,c) Quantification of downstream signaling is presented as the ratio of phosphorylated protein to GAPDH. (b) Normalized values for each variant compared to the wild type at 37°C. (c) Rations for each variant at 32°C versus 37°C. Symbols represent fold changes: “-” (≥ 30% decrease), “≈” (−30% to +50%), “(+)” (150–200%), “+” (2- to 4-fold), “++” (4- to 10-fold), “+++” (10- to 50-fold), “++++” (> 50-fold). Bar charts summarizing these data are provided in Figures S4 and S5.

The identification of specific tyrosine residue overactivation prompted an exploration into the temperature effects of the downstream signaling of these variants. To explore the molecular effects of p.Asn666 variants, we systematically examined the selected downstream signaling proteins of PDGFR*β* (Figure 3). At 37°C, all p.Asn666 variants exhibited an overactivating effect on one or more signaling mediators, namely, AKT, STAT1, STAT3, STAT6, and PLC*γ* (Figure 3a,b). Each p.Asn666 substitution demonstrated a distinct pattern. To illustrate, p.Asn666Lys displayed at least a twofold increased phosphorylation level of all examined downstream signaling proteins compared to wild type (Figure 3a,b). At 37°C, comparable levels of phosphorylated STAT1, AKT^Ser473^, and PLC*γ* were seen in p.Asn666Tyr and wild type, while AKT^Thr308^, STAT3, and STAT6 showed at least a fourfold increase. Downstream signaling of p.Asn666Ser and p.Asn666His exhibited similar patterns at 37°C, with at least twofold increase of phosphorylated AKT^Thr308^, STAT1, and STAT3 while maintaining phosphorylated AKT^Ser473^ and PLC*γ* levels similar to the wild type. Notably, while phosphorylated STAT6 was increased more than 10-fold in p.Asn666Lys, less than a twofold increase was seen in p.Asn666Ser compared to wild type (Figure 3a,b). These distinct phosphorylation patterns in downstream signaling indicate a different constitutively active gain-of-function pattern in all four variants.

We then investigated how downstream signaling proteins respond to a lower physiological temperature (Figure 3). We observed that the activation of the PI3K-AKT pathway was the dominant pattern in p.Asn666Tyr and p.Asn666Lys cells. A particularly prominent 32°C effect was observed for the p.Asn666Tyr substitution (Figure 3). On the other hand, upon exposure to 32°C, p.Asn666Ser, p.Asn666His, and p.Asn666Lys led to increased phosphorylation of various partners in the STAT pathway (Figure 3). At 32°C, the p.Asn666Ser variant exhibited a more than 10-fold increase in p-STAT1 indicating a strong activation of p-STAT1. The p.Asn666Lys substitution had, in addition to p-STAT1, an extensive increase of p-STAT6 at 32°C (more than 50-fold and 4-fold, respectively). For p.Asn666His, all three investigated STAT family members were overactivated at 32°C; p-STAT1 and p-STAT3 increased more than 50-fold, while at least a 10-fold increase was seen for p-STAT6. Notably, all four variants exhibited varying degrees of increased phosphorylation levels in the PLC*γ*1 pathway at 32°C (Figure 3).

### 3.4. The Temperature-Induced PDGFR*β* Downstream Signaling Kinetics in Temperature-Sensitive p.Asn666 *PDGFRB* Variants

In this section, we aimed to explore the dynamics of temperature-induced downstream signaling in PDGFR*β*. Specifically, we examined the time course of downstream signaling activation upon exposure to 32°C and the rate of normalization upon returning to 37°C after 1 h at 32°C.

The PI3K-AKT pathway exhibited a rapid response to temperature changes, with almost immediate increases observed after 5 min in p-AKT^Ser473^ and slightly longer, about 10 min, in p-AKT^Thr308^ for the p.Asn666Tyr substitution (Figure S6). In contrast, the STAT family members, p-STAT1, p-STAT3^Tyr-705^, and p-STAT6, displayed slower responses to temperature changes in all four p.Asn666 substitutions, typically requiring 30 min to 1 h for the activation (Figure S7). The 32°C-induced activation time for p-PLC*γ*1 was dependent on the PDGFR*β* amino acid substitution, ranging from 15 min for p.Asn666Lys to 1 h for p.Asn666Tyr, with a gradually accumulative trend for all four substitutions (Figure S8).

After exposing the p.Asn666Tyr substitution to 32°C for 1 h and returning to 37°C, we observed a rapid normalization of p-AKT^Ser473^, p-AKT^Thr308^, p-STAT1, p-STAT3, p-STAT6, and p-PLC*γ* levels (Figures S6, S7, and S8). Despite the observation of slower recovery kinetics for p.Asn666His, p.Asn666Ser, and p.Asn666Lys, it is noteworthy that temperature-induced activation is reversible in all four variants.

## 4. Discussion

The identification of five germline variants in the p.Asn666 position associated with severe clinical manifestations suggests that this position may play a crucial role in PDGFR*β* signaling and disease development. The p.Asn666Lys substitution in PDGFR*β* has been predicted to abolish the interaction between Asn666 and His661, changing potential protein interactions in this region and possibly leading to a structure more similar to the active conformation of KIT (proto-oncogene c-KIT) kinase [19]. The other disease-causing substitutions in neighboring *PDGFRB* sites further support that notion. The p.Val665Ala variant triggers constitutive activation of PDGFR*β* and is linked to Penttinen syndrome [20, 34, 35], while the loss-of-function p.Leu658Pro is associated with primary familial brain calcification [25, 36].

In this study, we confirmed that p.Asn666Tyr, p.Asn666Ser, p.Asn666His, and p.Asn666Lys are gain-of-function *PDGFRB* variants, causing constitutive ligand-independent activation [19, 21–23] (Figure 1). The total overactivation of PDGFR*β* itself could not account for the distinct and quite different, clinical manifestations seen in these p.Asn666 variants. We observed that p.Asn666Tyr, p.Asn666Lys, and p.Asn666His showed further increased total phosphorylation of PDGFR*β* at 32°C measured by ELISA, while p.Asn666Ser did not reach statistical significance (Figure 1). In addition, all variants exhibited temperature responses at specific PDGFR*β* tyrosine residues at 32°C (Figure 2), indicating their temperature-sensitive nature.

The phosphorylation pattern of specific tyrosine residues was observed to be different when cells were stimulated with ligand. The increased phosphorylation was predominantly observed in the higher 180–190 kDa band that represents the fully mature PDGFR*β* located in the plasma membrane [37, 38]. The receptor has to be situated in the plasma membrane for activation by extracellular ligands. In contrast, at 32°C, also the lower 160 kDa band showed increased phosphorylation (Figure 2). The 160 kDa band represents an immature form of PDGFR*β* [37, 38]. The ligand-dependent activation of the p.Asn666 variants thus revealed variant-specific phosphorylation of investigated tyrosine residues, highlighting distinctive phosphorylation patterns for each p.Asn666 variant (Figure 2).

At 37°C, all variants exhibited overactivation in specific downstream signaling pathways compared to the wild type, confirming their constitutively active nature (Figure 3a,b). At 32°C, an increased activation in various downstream signaling proteins was observed across all four p.Asn666 variants. The p.Asn666Tyr variant exhibited elevated levels of both p-AKT^Thr308^ and p-AKT^Ser473^ (Figure 3a,c), confirming original findings in patient fibroblasts [22]. Furthermore, the overactivation of STAT1 at 32°C was observed in all four variants, although this ratio might be less accurate in the p.Asn666Tyr variant, as the amount of p-STAT1 at 37°C approached background levels (Figure 3a,c). Such temperature-sensitive overactivation of phosphorylated p-STAT1 in the p.Asn666Ser variant was previously reported in patient fibroblasts [22].

It is thought that specific residues serve as a docking site for distinct downstream signaling proteins [5]. Our results only partly support this notion. For instance, the Y-751 residue serves as one of the docking sites for the PI3K-AKT signaling pathway [39, 40]. In line with this, we find that the p.Asn666Tyr variant exhibited the strongest activation of Y-751, resulting in a strong activation of both p-AKT^Ser473^ and p-AKT^Thr308^ at 32°C. However, the p.Asn666Lys variant displayed robust AKT signaling at 37°C, but the phosphorylation level of Y-751 was among the lowest observed among the p.Asn666 variants. A similar scenario was observed with the Y-1009 residue, a docking site for PLC*γ* signaling [41, 42]. The p.Asn666His and p.Asn666Tyr variants had the strongest phosphorylation of the Y-1009 at 32°C, resulting in noticeable activation of PLC*γ* (Figures 2 and 3a,c). Conversely, the p.Asn666Lys variant exhibited strong phosphorylation of PLC*γ* with undetectable levels of p-Y-1009 both at 37°C and 32°C (Figures 2 and 3a,c). This may suggest the presence of alternative docking sites for the downstream signaling pathways [43], or it could indicate variations in the timing between tyrosine residue phosphorylation and downstream signaling phosphorylation at different temperatures. These phosphorylation patterns represent a dynamic process captured at a specific time point under defined experimental conditions. This may contribute to the differences in observed phosphorylation peaks at individual PDGFR*β* tyrosine residues and in downstream signaling. Variability in the phospho-Y antibody affinity, specificity, and epitope accessibility may also affect the signal intensity and contribute to the consistently strong signals of p-Y-751 and p-Y-1009. Quantitative phospho-proteomics could provide an unbiased assessment of site-specific phosphorylation and clarify potential antibody-dependent variability.

AKT is involved in PDGFR*β*-mediated cellular processes, including proliferation, differentiation, survival, growth, migration, and angiogenesis [44, 45]. Furthermore, gene variants causing overactivation of the PI3K/AKT/mTOR pathway are implicated in overgrowth disorders [46]. The clinical manifestations of overgrowth observed in the p.Asn666Tyr variant, affecting the cornea and digits [22], are associated with increased phosphorylation of AKT at 32°C (Figure 3; Figure S6). A strong AKT involvement at both temperatures was observed in the p.Asn666Lys variant (Figure 3), which could be associated with tumor overgrowth [47–49].

The STAT1 upregulation in PDGFR*β* signaling has been associated with a tissue-wasting phenotype [50]. Although speculative, the severe acro-osteolysis and tissue wasting of limbs described in the p.Asn666Ser variant and the less severe acro-osteolysis of hands in the p.Asn666His variant [21, 23, 31] (Table S1) could be linked to strong STAT1 upregulation at 32°C (Figure 3a). In contrast, the overgrowth phenotype in the p.Asn666Tyr variant exhibited low levels of STAT1 signaling at both temperatures (Figures 3a, 3b, and 3c).

The transcription factors STAT3 and STAT6 regulate cell functions like growth, differentiation, and immune responses, with aberrations in their activation linked to cancer, autoimmunity, and inflammation [51–54]. Similarly, PLC*γ*, a key enzyme in phosphoinositide signaling, influences cell growth, calcium regulation, and inflammation [55, 56]. A full understanding of the precise roles of PDGFR*β* downstream pathways in the development of diseases associated with p.Asn666 variants remains elusive.

Temperature kinetic studies indicate that temperature-induced ligand-independent phosphorylation of all four p.Asn666 *PDGFRB* variants is a dynamic and reversible process (Figure S6, S7, and S8). This dynamic nature and reversibility of temperature-induced activation adds further complexity to their downstream signaling. This observation prompts the consideration of potential mechanisms underlying the temperature sensitivity of the mutant kinase domain. This may indicate changes in intrinsic stability or efficiency at lower temperatures, or it may be due to indirect effects on phosphorylation regulators like phosphatases or processes such as endocytosis and membrane trafficking. Further research is needed to clarify these interactions and their impact on kinase function at varied temperatures.

## 5. Conclusions

Understanding the precise contributions of downstream signaling proteins to the clinical manifestations of the four p.Asn666 *PDGFRB* variants is a complex challenge. However, potential associations have been identified, such as that AKT signaling could influencing overgrowth phenotypes and STAT1 upregulation could be linked to tissue wasting or degenerative clinical features. The distinct pattern of phosphorylation of specific tyrosine residues of PDGFR*β* observed in the p.Asn666 variants provides a new possible explanation of how gain-of-function *PDGFRB* variants result in such strikingly different phenotypes. The four p.Asn666 *PDGFRB* variants exhibit variant-specific tyrosine residue and downstream signaling overactivation at 32°C, emphasizing the importance of temperature as an environmental factor.

## Data Availability

All data generated or analyzed during this study are included in this published article and its supporting information files.

## Supporting Information

Supporting Information Additional supporting information can be found online in the Supporting Information section. Table S1. Summary of clinical manifestations associated with five p.Asn666 PDGFRB variants, including variant names, phenotypes, and key clinical manifestations from previously published studies. The abbreviation “NR” indicates that information was not reported. Figure S1. Full immunoblotting images corresponding to the data presented in Figure 2. Figure S2. Overexposed immunoblotting images corresponding to the data presented in Figure 3. Figure S3. Full immunoblotting images corresponding to the data presented in Figure 3. Figure S4. Quantification analysis of the western blots presented in Figure 2, Panel B. The data shows the relative intensity of the protein bands normalized to the control. Figure S5. Quantification analysis of the western blots presented in Figure 2, Panel C. The data shows the relative intensity of the protein bands normalized to the control. Figure S6. Representative images showing the activation and subsequent normalization of p-AKT^Thr308^ and p-AKT^Ser473^ signaling in PDGFRB variants at 32°C. Left: Activation kinetics across time points (5 min to 6 h). Right: Normalization of p-AKT levels after returning to 37°C following 1 h at 32°C. Figure S7. Representative images showing the activation and normalization of p-STAT1, p-STAT3^Tyr705^, and p-STAT6 signaling in PDGFRB variants at 32°C. Left: Activation kinetics across time points (5 min to 6 h). Right: Normalization of p-STAT levels after returning to 37°C following 1 h at 32°C. Figure S8. Representative images showing the activation and normalization of p-PLC*γ*1 signaling in PDGFRB variants at 32°C. Left: Activation kinetics across time points (5 min to 6 h). Right: Normalization of p-PLC*γ*1 levels after returning to 37°C following 1 h at 32°C.
