# Supplementary material for: Temperature as a Key Modulator: Investigating Phosphorylation Patterns of p.Asn666 PDGFRB Variants and Their Role in Downstream Signaling
Source: Hum Mutat. 2025 Apr 22;2025:6664372. doi: 10.1155/humu/6664372 (PMC12041633; doi:10.1155/humu/6664372)
Supplement: Supporting Information — Additional supporting information can be found online in the Supporting Information section. Table S1. Summary of clinical manifestations associated with five p.Asn666 PDGFRB variants, including variant names, phenotypes, and key clinical manifestations from previously published studies. The abbreviation “NR” indicates that information was not reported. Figure S1. Full immunoblotting images corresponding to the data presented in Figure 2. Figure S2. Overexposed immunoblotting images corresponding to the data presented in Figure 3. Figure S3. Full immunoblotting images corresponding to the data presented in Figure 3. Figure S4. Quantification analysis of the western blots presented in Figure 2, Panel B. The data shows the relative intensity of the protein bands normalized to the control. Figure S5. Quantification analysis of the western blots presented in Figure 2, Panel C. The data shows the relative intensity of the protein bands normalized to the control. Figure S6. Representative images showing the activation and subsequent normalization of p-AKTThr308 and p-AKTSer473 signaling in PDGFRB variants at 32°C. Left: Activation kinetics across time points (5 min to 6 h). Right: Normalization of p-AKT levels after returning to 37°C following 1 h at 32°C. Figure S7. Representative images showing the activation and normalization of p-STAT1, p-STAT3Tyr705, and p-STAT6 signaling in PDGFRB variants at 32°C. Left: Activation kinetics across time points (5 min to 6 h). Right: Normalization of p-STAT levels after returning to 37°C following 1 h at 32°C. Figure S8. Representative images showing the activation and normalization of p-PLCγ1 signaling in PDGFRB variants at 32°C. Left: Activation kinetics across time points (5 min to 6 h). Right: Normalization of p-PLCγ1 levels after returning to 37°C following 1 h at 32°C. [file 6664372.f1.pdf]

Supplementary Materials

	**Individual 1**	**Individual 2**	**Individual 3**	**Individual 4**	**Individual 5**	**Individual 6**	**Individual 7**	**Individual 8**	**Individual 9**
Described by	Cheung et al.	Pond et al.	Bredrup et al.	Bredrup et al.	Aggarwal et al.	Bredrup et al.	Bredrup et al.	Bredrup et al.	Pattisapu et al.
Familial status	singleton individual	singleton individual	singleton individual 1	singleton individual 2	singleton individual	index mother	child of individual 6	child of individual 6	singleton individual
PDGFRB variant	p.Asn666Lys	p.Asn666His	p.Asn666Ser	p.Asn666Ser	p.Asn666Ser	p.Asn666Tyr	p.Asn666Tyr	p.Asn666Tyr	p.Asn666Thr
Mutation origin	somatic	germline	germline	germline	germline	germline	germline	germline	somatic
Stature	NR	Tall	Tall	Tall	Tall	Normal	Normal	Normal	NR
Keloid (hands and feet)	NR	NR	-	-	NR	+	+	+	NR
Corneal vascularization / Pterygium	NR	-	+	+	+	+	+	+	NR
Secondary exophthalmos	NR	-	+	+	+	-	-	-	NR
Reduced vision	NR	-	+	+	+	+	+	+	NR
Thin, translucent skin	NR	+	+	+	+	-	-	-	NR
Skin ulceration	NR	-	+	+	-	-	-	-	NR
Abnormal skin pigmentation	NR	-	-	+	+	-	+	+	NR
Lipodystrophy	NR	-	+	+	+	-	-	-	NR
Acro-osteolysis	NR	+	+	+	+	-	-	-	NR
Joints contractures	NR	+	+	+	+	+	+	+	NR
Short, broad digits	NR	+	+	+	+	NR	NR	NR	NR
Camptodactyly finger	NR	+	+	+	+	+	+	+	NR
Camptodactyly toes	NR	NR	+	+	+	-	-	-	NR
Nasal bridge	NR	broad	narrow	narrow	NR	NR	NR	NR	NR
(Pre)maxilla	NR	NR	+	+	+	-	-	-	NR
Thin upper Vermilion	NR	NR	+	+	+	-	-	-	NR
Kyphosis	NR	NR	NR	NR	+	-	-	-	NR
Scoliosis	NR	NR	+	+	+	-	-	-	NR
Fibroma	Myofibromas in abdominal wall and left upper gingival border (somatic p.Asn666Lys, and germline p.Arg561Cys variants), myofibroma in neck and blood (somatic p.Arg561Cys *PDGFRB* variant)	Orbital wall dermoid cyst that spontaneously resolved (myofibroma?)	-	-	-	-	-	-	Prenatally diagnosed, large, solid, partially necrotic right facial myofibroma; with focal skeletal lucencies (somatic p.Asn666Thr and p.Ile564_Val572del)
Hemangioma	NR	+	+	+	-	-	-	-	NR
Craniosynostosis	NR	+	NR	NR	+	-	-	-	NR
Hydrocephalus	NR	?	+	+	-	-	-	-	NR
Progeria-like aging	NR	-	+	+	+	-	-	-	NR
Mental status	NR	Normal	Normal	Normal	Normal	Normal	Normal	Normal	NR
Other phenotype features	-	Coarse facial features, intracranial cysts, delayed teeth eruption, gingival hypertrophy, carpal tunnel syndrome	Delayed teeth eruption	-	Subdural hygroma, delayed teeth eruptiop, abnormal teeth, prolonged face	-	-	-	-

***Supplementary Table 1**. Summary of clinical manifestations associated with five p.Asn666 PDGFRB variants, including variant names, phenotypes, and key clinical manifestations from previously published studies. The abbreviation “NR” indicates that information was not reported.*

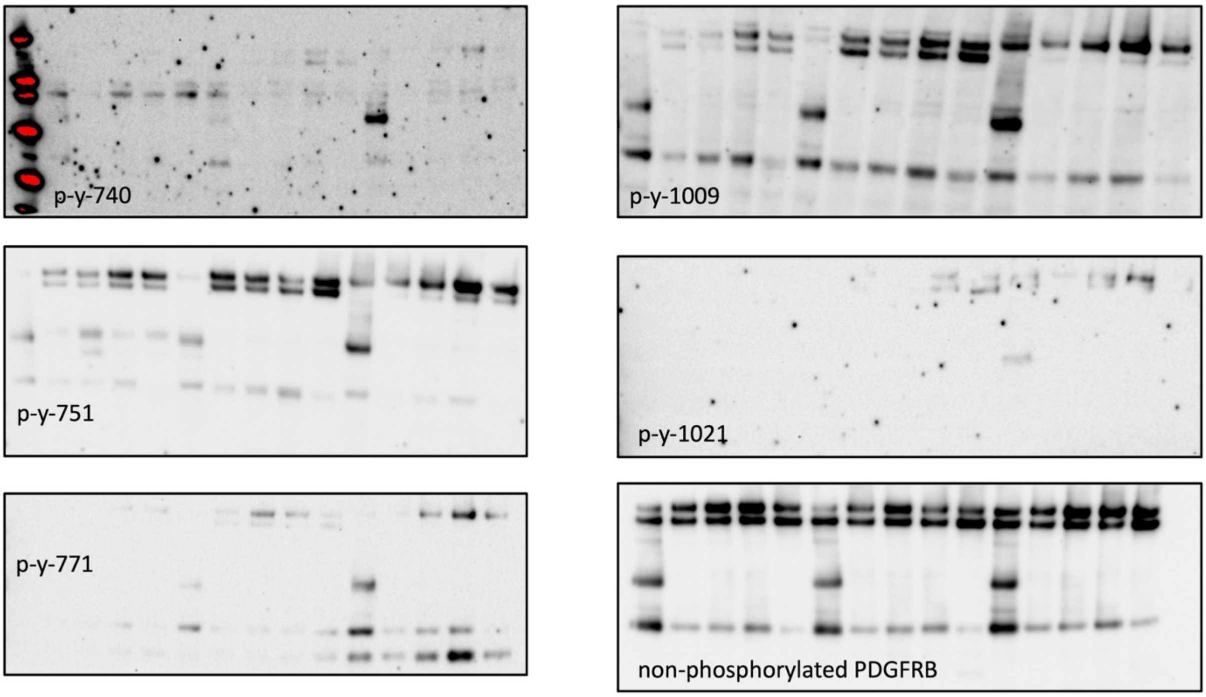

Supplementary Figure 1. Full immunoblotting images corresponding to the data presented in Figure 2.

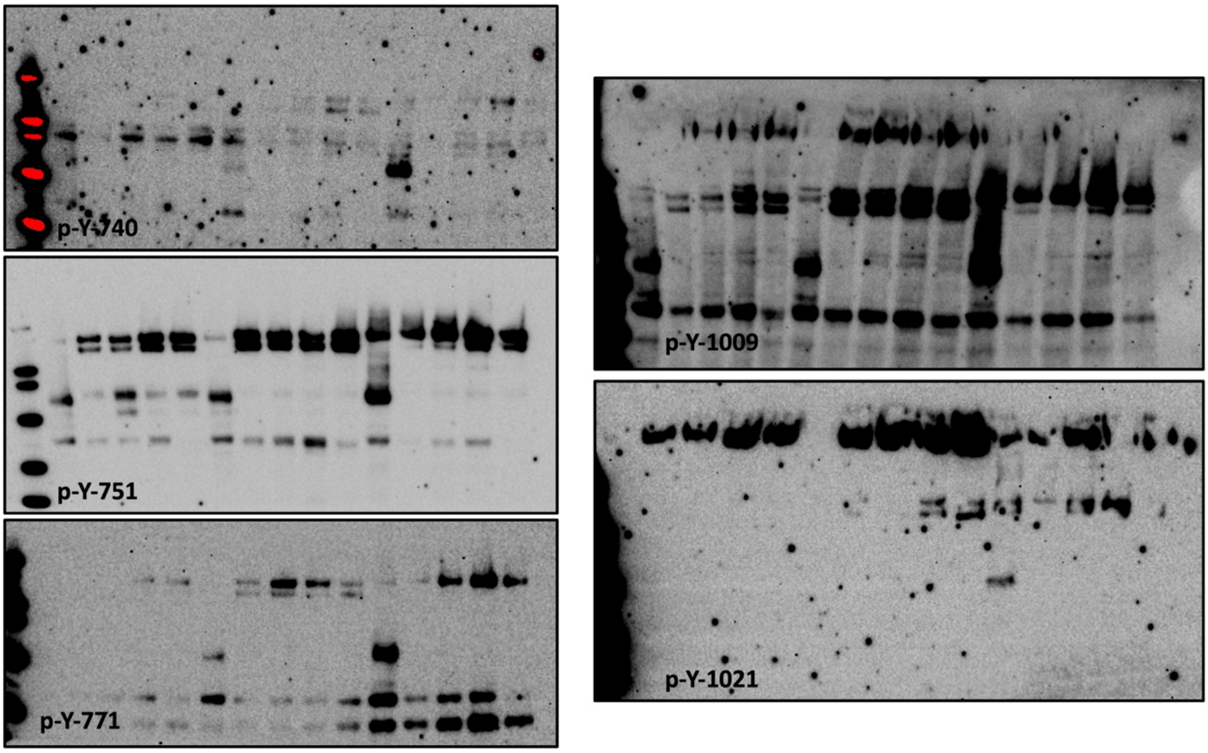

Supplementary Figure 2. Overexposed immunoblotting images corresponding to the data presented in Figure 2.

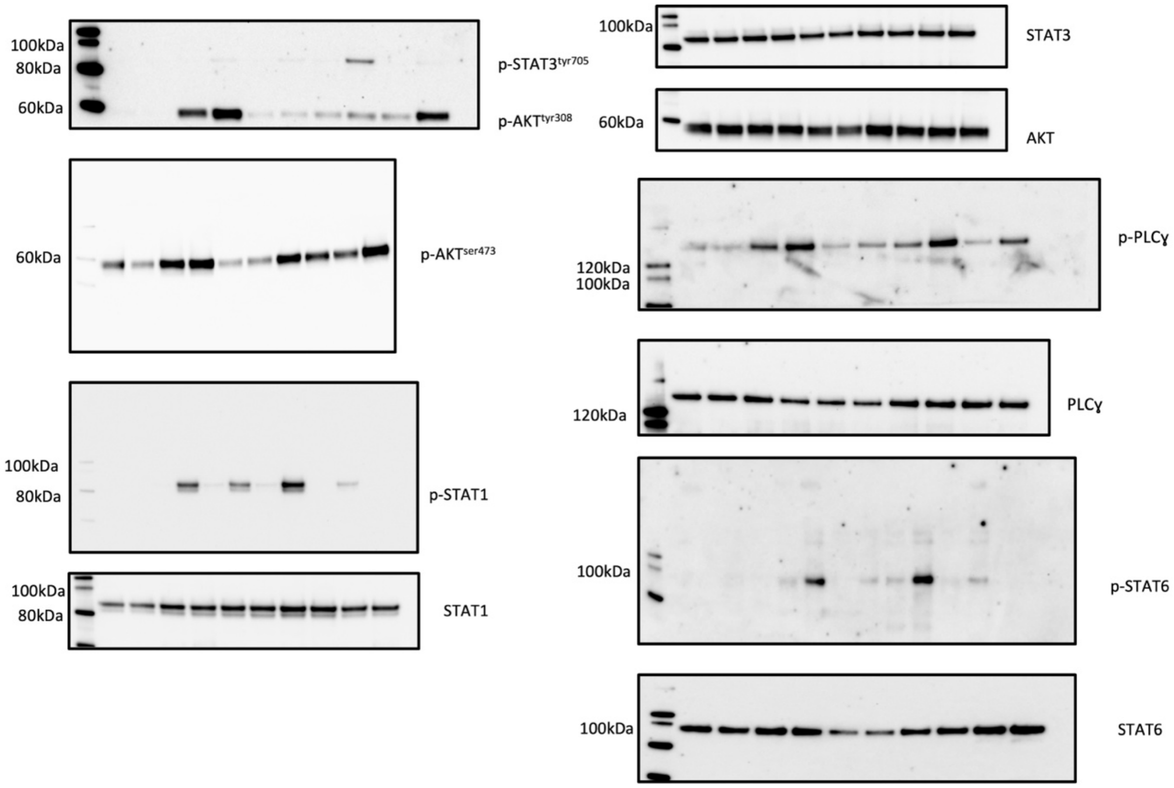

Supplementary Figure 3. Full immunoblotting images corresponding to the data presented in Figure 3, Panel A.

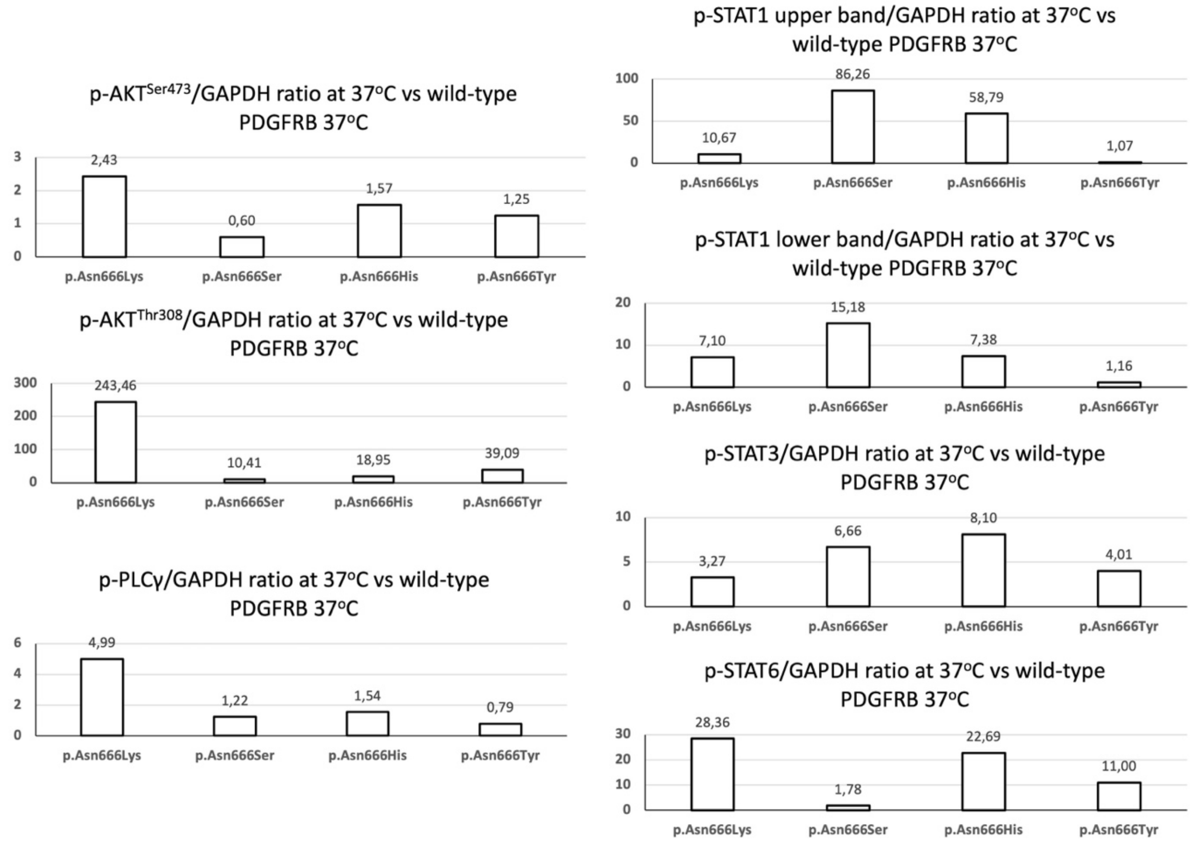

Supplementary Figure 4. Quantification analysis of the Western blots presented in Figure 3, Panel B. The data shows the relative intensity of the protein bands normalized to the control.

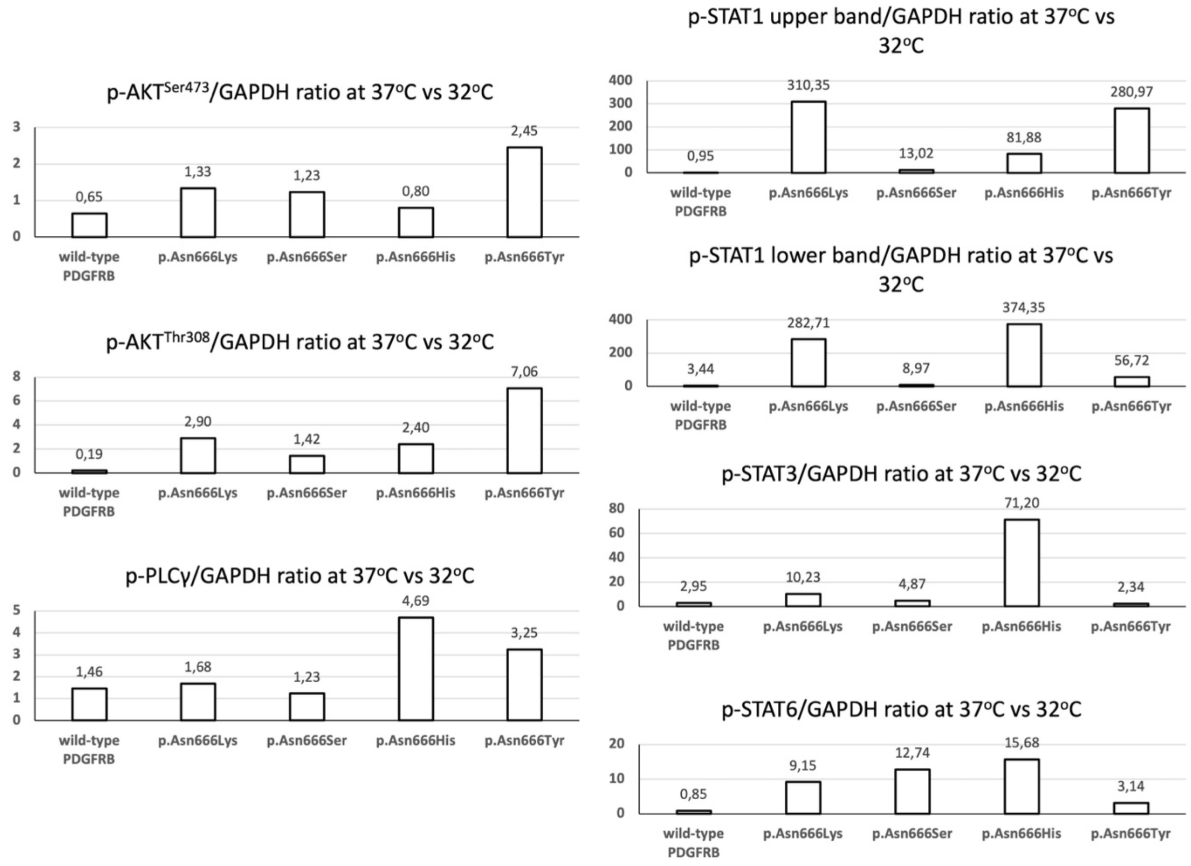

Supplementary Figure 5. Quantification analysis of the Western blots presented in Figure 3, Panel C. The data shows the relative intensity of the protein bands normalized to the control.

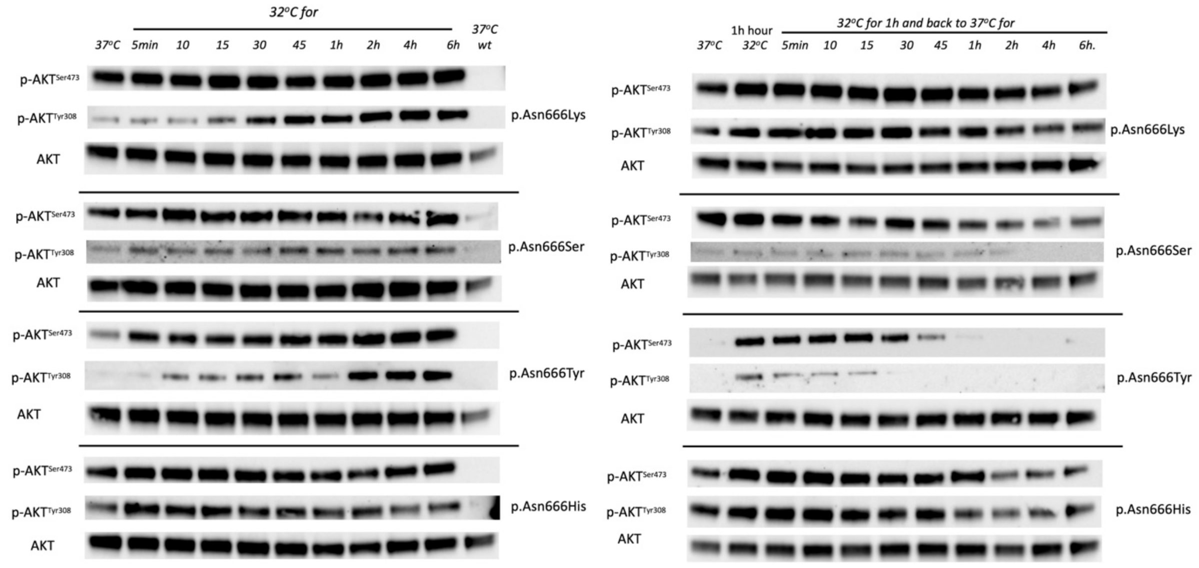

Supplementary Figure 6. Representative images showing the activation and subsequent normalization of p-AKT^{Thr308} and p-AKT^{Ser473} signaling in PDGFRB variants at 32°C. Left: Activation kinetics across time points (5 minutes to 6 hours). Right: Normalization of p-AKT levels after returning to 37°C following 1 hour at 32°C.

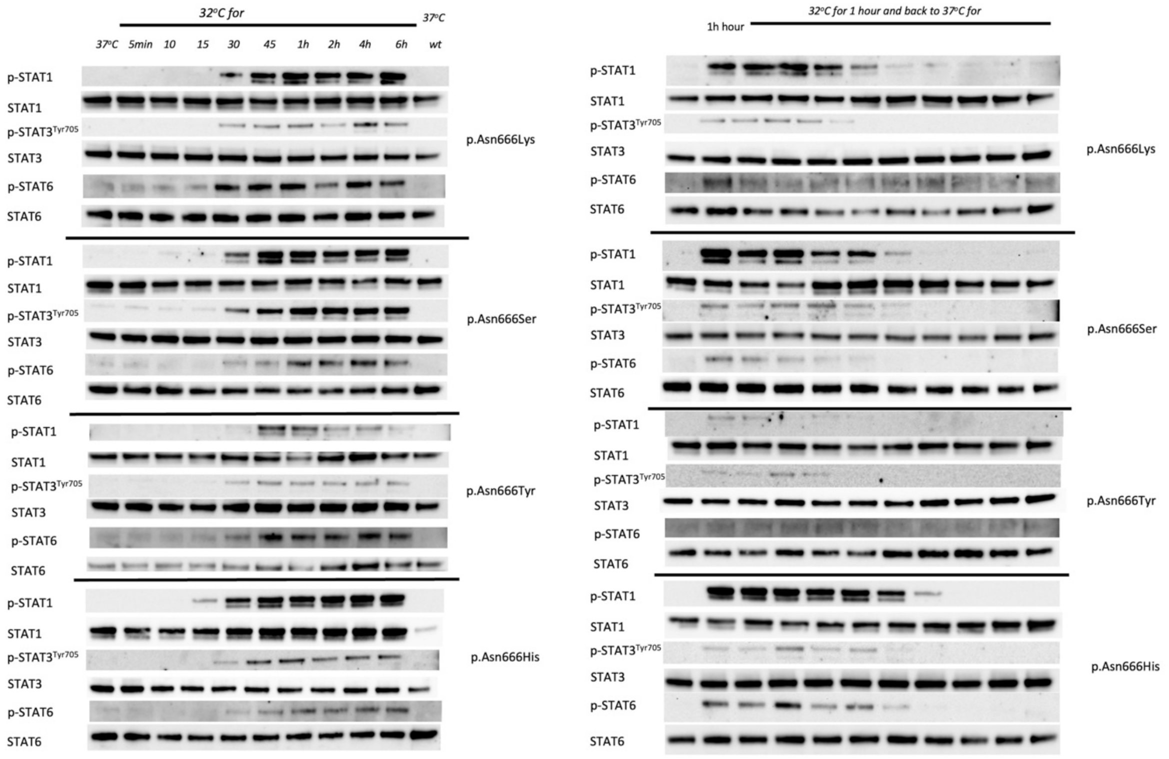

Supplementary Figure 7. Representative images showing activation and normalization of p-STAT1, p-STAT3^{Tyr705}, and p-STAT6 signaling in PDGFRB variants at 32°C. Left: Activation kinetics across time points (5 minutes to 6 hours). Right: Normalization of p-STAT levels after returning to 37°C following 1 hour at 32°C.

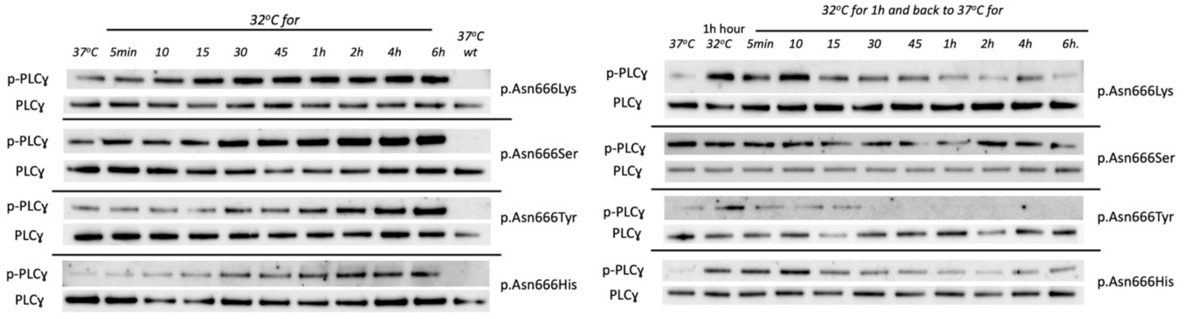

Supplementary Figure 8. Representative images showing activation and normalization of p-PLCγ1 signaling in PDGFRB variants at 32°C. Left: Activation kinetics across time points (5 minutes to 6 hours). Right: Normalization of p-PLCγ1 levels after returning to 37°C following 1 hour at 32°C.
